# Cohort Profile: The Northern Ireland Cohort for the Longitudinal Study of Ageing (NICOLA)

**DOI:** 10.1093/ije/dyad026

**Published:** 2023-04-03

**Authors:** Charlotte Neville, Frances Burns, Sharon Cruise, Angela Scott, Dermot O’Reilly, Frank Kee, Ian Young

**Affiliations:** Centre for Public Health, School of Medicine, Dentistry and Biomedical Sciences, Queen’s University Belfast, Belfast BT12 6BJ, UK; Centre for Public Health, School of Medicine, Dentistry and Biomedical Sciences, Queen’s University Belfast, Belfast BT12 6BJ, UK; Centre for Public Health, School of Medicine, Dentistry and Biomedical Sciences, Queen’s University Belfast, Belfast BT12 6BJ, UK; Centre for Public Health, School of Medicine, Dentistry and Biomedical Sciences, Queen’s University Belfast, Belfast BT12 6BJ, UK; Centre for Public Health, School of Medicine, Dentistry and Biomedical Sciences, Queen’s University Belfast, Belfast BT12 6BJ, UK; Centre for Public Health, School of Medicine, Dentistry and Biomedical Sciences, Queen’s University Belfast, Belfast BT12 6BJ, UK; Centre for Public Health, School of Medicine, Dentistry and Biomedical Sciences, Queen’s University Belfast, Belfast BT12 6BJ, UK

Key Features

- The Northern Ireland Cohort for the Longitudinal Study of Ageing (NICOLA) is Northern Ireland’s largest health and social care cohort collecting longitudinal data from a representative sample of the over-50s population.
- 8283 participants aged ≥50 years and living in private residential accommodation were recruited, from a randomized, stratified sample of Northern Ireland addresses, to the Wave 1 cohort between December 2013 and March 2016. A follow-up (Wave 2) of the cohort took place between May 2017 and November 2019, with a response rate of 73% (*n* = 6152).
- NICOLA participants will undertake a detailed survey, in their own home, every 2 years with a detailed health assessment every 4 years.
- The NICOLA data set comprises a diverse range of objective and subjective measures of physical and mental health, life expectancy, disability, education, economic activity, social participation and support, household and family structures; and uniquely to NICOLA, the effects of ‘the Troubles’, a detailed ophthalmological assessment and a dietary assessment. Inclusion of unique measures allows analysis of the social and contextual influences on the distribution of health status determinants particular to Northern Ireland.
- The collection of data via subjective reporting in interviews and self-completion methodologies combined with objective measurement of health and wellbeing allows detailed analysis of the ageing profiles.
- The NICOLA cohort is flagged on the National Health Applications Infrastructure Service to enable robust and comprehensive linkage for identification of participant outcomes and affecting factors.
- The harmonization of health status measures with other ageing cohorts allows cross cohort comparative analysis, those of particular relevance being the English Longitudinal Study of Ageing (England) and The Irish Longitudinal Study of Ageing (Republic of Ireland). Comparability of NICOLA to other ageing studies will also be enabled via the Gateway to Global Aging Data (G2G) and with NICOLA data being archived within the Dementias Platform UK (https://www.dementiasplatform.uk/research-hub/data-portal).
- Access to the anonymized data is by direct application to the NICOLA study (https://nicola.qub.ac.uk/sites/NICOLA/InformationforResearchers/#requesting-access-to-nicola-data-or-biological-samples-910951-1).

## Why was the cohort set up?

Although a region of the UK previously known for having a younger population, Northern Ireland (NI) is experiencing a similar demographic transition that is seeing life expectancy continuing to rise. Government population projections^1^ indicate that by 2028 39% of the population will be ≥50 years of age, with this statistic rising to 43% by 2048. This increase in the ageing population presents challenges, particularly for policy makers wishing to support citizens through the equitable provision of health and social care services and wider economic policies.

NICOLA closely follows the comprehensive approaches taken by the English Longitudinal Study of Ageing (ELSA)^2^, established in 2002, and the Republic of Ireland’s Irish Longitudinal Study on Ageing (TILDA)^3^ established in 2009. These, and other international longitudinal cohort studies of ageing, can yield much that could be useful to policy makers. However, NI has a unique social history, relating specifically to political and social unrest, justifying closer scrutiny in a local cohort. To reflect this, NICOLA has particular focus on intergenerational poverty, transition points in ageing and the effects of diet on the ageing process. The study also includes questions of unique relevance to ‘the Troubles’—a time of political violence in NI between the late 1960s and the late 1990s, and its consequences for the ageing NI population.

NICOLA is supported through a portfolio of funders that recognized the need for a Northern Irish ageing cohort. The Atlantic Philanthropies (AP) indicated its early support for the cohort and shortly thereafter the Northern Ireland Executive Office committed funding for the NICOLA pilot study in 2012. Following the successful pilot, the UK Clinical Research Collaboration (UKCRC), the Economic and Social Research Council, the Centre for Ageing Research and Development in Ireland (CARDI) and the Northern Ireland Public Health Agency Research and Development office (HSC R&D) added to the AP funding to ensure a successful launch of NICOLA. NICOLA is based within the Centre for Public Health, Queen’s University Belfast, with a Stakeholder Council with representation from: the Northern Ireland Executive Office, the devolved Northern Ireland government department, within the Northern Ireland Executive; a range of Executive Agencies including the Department for Communities and Department for the Economy; the local Health and Social Care (HSC) infrastructure; the Institute of Public Health Ireland and The Irish Longitudinal Study of Ageing (TILDA).

As with other cohorts, NICOLA focuses on the various aspects of the ageing process and how they interact. To this purpose, the involvement of expertise from medical and health sciences, economics and management, social sciences and psychology within NICOLA aims to maximize the advantage of multidisciplinary research, both within Queen’s University Belfast and through extensive collaboration with international research groups. NICOLA is closely affiliated to the Administrative Data Research Centre Northern Ireland (ADRC-NI)^4^ and will maximize the cohort data through ethically approved linkages to available administrative data sets. Data linkage will include, in the first instance, linking the NICOLA data to routinely collected health and social care data from the Business Service Organisation (BSO) Honest Broker Service (HBS)^5^ and also linkage with the Northern Ireland Cancer Registry^6^. Examples of such data include hospital admissions, general practitioner and dental records, health screening registers and disease registers.

## Who is in the cohort?

NICOLA is a longitudinal panel of 8283 participants representative of an ‘eligible population’ defined as community dwelling adults aged ≥50 years living in private households in NI, broadly equivalent in sample size to both TILDA^3^ and ELSA^2^. Those living in residential care homes or other institutionalized settings, e.g. nursing homes, or who lacked the capacity to provide informed consent were not eligible to participate. Spouses or partners of eligible participants who shared the same residency were also invited to participate, regardless of their age. The spouse or partner completed the same assessments. The purpose of including the spouse or partner was to obtain overall household-level information including, for example, finances and household structure. Other members of the household were not included. A total of 195 who were aged <50 years chose to participate, resulting in a total of 8478 participants in NICOLA Wave 1.

The BSO General Practitioner Register Database was used as the source for identifying addresses containing eligible individuals. The sample was ordered using geographic stratification, identified addresses ordered within postcode geography and then a fixed interval (systematic) sample drawn. The sample was merged with the Pointer Database of addresses for NI maintained by Land and Property Services providing a common standard address for every property, enabling the full range of address information to be available for fieldwork interviewers to plan their schedule of work. All individuals living at each address were included in the sample. NICOLA required a total of 12 077 addresses (main sample) and in addition a 20% sample contingency of 2415 addresses (reserve sample), resulting in a total of 14 492 addresses in the initial sampling frame. Both the main and reserve samples were extracted at the start of the project. In a final addition, a further sample of 2416 addresses (additional reserve) were extracted from BSO in the later stages of Wave 1 fieldwork to enable the target cohort number to be achieved. The purpose of the reserve samples was to compensate for potentially lower-than-expected response rates to the initial study invitation. In total, 16 908 households received an invitation letter to take part in Wave 1.

To be eligible to take part in NICOLA, individuals must be aged ≥50 years and therefore born on or before 30 September 1962. All individuals aged ≥50 years enumerated in respondent sample households (*n* = 16 908 households) were invited to become part of the longitudinal sample. An introductory letter was sent to the household (unnamed—householder only rather than a specific individual). The household was then visited by an Ipsos MORI fieldworker who obtained the name and informed consent from eligible participants. Individuals not yet 50 years of age, who were institutionalized or who lacked the capacity to provide informed consent were excluded from joining NICOLA. The NICOLA study was designed to follow similar sampling protocols to those used in TILDA^3^ and ELSA^2^, thus facilitating comparative analysis. As with ELSA and TILDA, participants were selected according to a community home address rather than a nursing or residential home. In addition, the governance implications of accessing individuals in institutionalized care was considered to be too challenging in terms of time, resources and impact on overall recruitment rates. The general health status of many individuals in care settings may also have affected their ability to both consent and participate in the computer-assisted personal interview (CAPI) and health assessment. Based on this, it was decided to only recruit individuals living in the community.

Partners of eligible participants did not have to have been born before 30 September 1962 but had to be living at the same address as the selected age-eligible participant. As part of the written consent process, participants were asked whether they agreed to future contact by the NICOLA study team. It is the expectation that, within future waves, participants will be followed up for admission into residential institutions, depending on ethical approval.

## How often will they be followed up?

NICOLA aims to collect data from all participants within structured waves of ∼2-year intervals. The proposed design of Wave 3 (pending funding) will include a home-based health assessment, CAPI and self-completion questionnaire (SCQ) with a focus on the following research topics: immune response, the microbiome, digital inclusion, food insecurity, cognitive health and eye health.

With continued follow-up, all NICOLA participants will be invited to take part in a CAPI every 2 years and a detailed health assessment every ∼4 years. The use of the SCQ is intended as an adjunct within each wave. It is the intention that participants will be followed up for a period of ≥10 years.

## What has been measured?

### NICOLA Wave 1

The Central Survey Unit of the Northern Ireland Statistics and Research Agency (NISRA)^7^ was commissioned by the NICOLA project team to conduct an initial pilot of the CAPI on 197 addresses. Of these 197, 186 addresses were eligible (i.e. one household member living at the address was aged ≥50 years) and, of these, 89 households consented to participate (*n* = 153 participants), thus representing a 48% household response rate. The pilot study was conducted between October and November 2013. The main survey was informed by this short pilot study with a revised format, content and length, CAPI questionnaire and supplementary SCQ utilized in Wave 1 NICOLA fieldwork (see Table 1).

**Table 1:** Summary of data collected in NICOLA^a^ Wave 1 and Wave 2

	Wave 1	Wave 2
Demographics		
Household membership	**√**	**√**
Living relatives	**√**	**√**
Marital status	**√**	**√**
Ethnic group	**√**	**√**
Country of birth	**√**	**√**
Education	**√**	**√**
Occupation of main carer when respondent was 14 years old	**√**	**√**
Parent status and history		**√**
Transfers to children		
Non-financial assistance to children	**√**	**√**
Physical and cognitive health		
Self-reported physical and mental health^b^	**√**	**√**
Activities of daily living	**√**	**√**
Brief resilience scale	**√**	**√**
Mini mental state examination (MMSE)	**√**	**√**
Loneliness scale	**√**	**√**
Pets	**√**	**√**
General practitioner visits	**√**	**√**
Memory recall	**√**	**√**
Word recall	**√**	**√**
Physician diagnosed conditions	**√**	**√**
Chronic conditions	**√**	**√**
Cancer screening	**√**	**√**
Falls/fear of falls/balance/fractures	**√**	**√**
Ophthalmology/visual disability^b^	**√**	**√**
Pain	**√**	**√**
Incontinence	**√**	**√**
Menopause	**√**	**√**
Health and disability vignettes^b^	**√**	**√**
Oral health		**√**
Disability^b^		**√**
Hospital anxiety and depression scale (HADS)^b^		**√**
CASP-19 (quality of life)		**√**
Quality of life (WHOQOL-BREF)^b^		**√**
Life events, trauma and stress		
The Troubles^b^	**√**	
Post-traumatic stress^b^	**√**	
Personality		
Risk perception^b^	**√**	
Trust^b^	**√**	
Optimism	**√**	
Personality measure (NEO Five Factory Inventory)^b^	**√**	
Help and helpers		
Activities of daily life	**√**	**√**
Help and helpers	**√**	**√**
Employment		
Employment/unemployment situation	**√**	**√**
Job details	**√**	**√**
Subsidiary work		**√**
Work stress	**√**	**√**
Retirement and reason for retirement	**√**	**√**
Unpaid work/caring	**√**	**√**
Working conditions/job satisfaction		**√**
Healthcare utilization		
General practitioner/Accident and Emergency	**√**	**√**
Hospital outpatient/day procedure/overnight	**√**	**√**
Satisfaction with Accident and Emergency service		**√**
Reason for visit to Accident and Emergency		**√**
Outcome of visit to Accident and Emergency		**√**
Use of publically funded health service		**√**
Satisfaction with publically funded health service		**√**
Health behaviour		
Smoking/smoking history	**√**	**√**
E-cigarette use		**√**
Alcohol consumption	**√**	**√**
Sleep duration and sleep disturbance	**√**	**√**
Physical activity^b^	**√**	**√**
Partner sexual/romantic intimacy		**√**
Medications		
Polypharmacy	**√**	**√**
Work and pensions		
Employment and state pensions	**√**	**√**
Retirement age	**√**	**√**
Pension contributions and payments	**√**	**√**
Social connectedness		
Connectedness to family and non-family structures	**√**	**√**
Religious affiliation	**√**	**√**
Computer and internet use^b^	**√**	**√**
Involvement in groups, clubs, volunteering	**√**	**√**
Culture and arts activities^b^		**√**
Income and assets		
Earnings	**√**	**√**
Sources of income	**√**	**√**
Pension	**√**	**√**
Benefit entitlement and receipt	**√**	**√**
Savings/stocks, shares and investments	**√**	**√**
Financial and physical assets	**√**	**√**
Life insurance	**√**	**√**
Inheritance	**√**	**√**
Debt	**√**	**√**
Expectations		
Financial situation and comparisons	**√**	**√**
Social participation		
Accommodation	**√**	**√**
Local area perception	**√**	**√**
Housing		**√**
Transport		**√**
Social capital		
Views about residential area	**√**	**√**
Neighbourhood perception	**√**	**√**
Social networks	**√**	**√**
Social support	**√**	**√**
Social and cultural participation	**√**	**√**
Unpaid help	**√**	**√**
HEALTH ASSESSMENT		
Cognition and mental health		
Mini Mental State Examination (MMSE)	**√**	**√^c^**
Colour trails 2	**√**	
Montreal Cognitive Assessment (MOCA)	**√**	
Animal recall	**√**	
Center for Epidemiological Studies Depression (CES-D)	**√**	
Warwick Edinburgh Mental Wellbeing Scale (WEMWBS)^b^	**√**	
Cardiovascular and muscular		
Blood pressure	**√**	
Grip strength	**√**	
Anthropometric measures	**√**	
Body composition	**√**	
Gait and balance		
Step test	**√**	
Timed ‘up and go’	**√**	
Respiratory		
Spirometry	**√**	
Photography		
Detailed facial imaging	**√**	
Blood and urine		
Non-fasting samples	**√**	
Ophthalmic and hearing		
Visual function	**√**	
Refractive status	**√**	
Intra ocular pressure	**√**	
Ophthalmic retina imaging	**√**	
Hearing	**√**	
Nutrition^d^		
Food frequency questionnaire	**√**	
Dietary supplements	**√**	
Allergies or food-related health issues	**√**	
Specified diets	**√**	
Shopping	**√**	
Cooking	**√**	
End-of-life questionnaire		
Cause of death		**√**
Residence prior to death		**√**
I(ADL) and helpers		**√**
Physical health		**√**
Behavioural health		**√**
Cognitive function		**√**
Mood		**√**
Healthcare utilization including costs		**√**
Assets and life insurance		**√**
COVID-19 questionnaire^b^		
Physical health		**√**
Mental health		**√**
Food provision		**√**
Financial security		**√**
Work and finances		**√**
Volunteering and caring		**√**
Lifestyle		**√**
Social connections		**√**
Pensions and retirement		**√**

a NICOLA, Northern Ireland Cohort for the Longitudinal Study of Ageing.

b Self-completion questionnaire.

c Completed as part of Wave 2 computer-assisted personal interview.

d Dietary self-completion questionnaire.

Wave 1 also includes a wide-ranging health assessment module conducted by trained registered nurses in the Wellcome Trust–Wolfson Northern Ireland Clinical Research Facility (NICRF) located in Belfast or, when necessary, in the participant’s home (see Table 1). Biological samples including a non-fasting blood and urine sample were collected from participants as part of the health assessment. All samples were initially processed in the NICRF within 4 h of phlebotomy. Once processed, the aliquots were stored short term in the freezers within the NICRF and then transported to the main laboratory in the Centre for Public Health at Queen’s University Belfast on a weekly basis. All biological samples were transported to the main laboratory in an insulated polystyrene box containing frozen cool packs. In the case of home visits, a dedicated courier service was used for transporting samples from the participant’s home to the main laboratory for processing. All aliquots were then stored in –80^o^C freezers until analysis. Samples were analysed for multi-omic biomarkers, lipids, dietary biomarkers, inflammatory biomarkers and hormones. Laboratory assays were standardized against available international standards, with quality-control samples included in every run. All biochemical biomarkers were analysed using the Abbott ARCHITECT c8000 system with the exception of testosterone, sex hormone binding globulin (SH-BG) and vitamin D, which were analysed using the Abbott ARCHITECT i2000 system. Methylation status was measured using the Infinium Methylation EPIC Bead Chip Array (Illumina USA). The health assessments were conducted between January 2014 and August 2018, whereas the CAPI interviews were completed between December 2013 and March 2016. The time frame between the CAPI interview and health assessment was 78 days (40, 322) [median (interquartile range)]. Further details regarding the methods used in the health assessment and the findings from the health assessment will be presented in a forthcoming manuscript. Key findings from the health assessment are also available in the NICOLA health assessment report, which can be accessed via https://www.qub.ac.uk/sites/NICOLA/ReportsandNewsletters/.

### Embedded randomized–controlled trials of study design

NICOLA Wave 1 included two randomized–controlled trials (RCTs) within its study design. The first of these aimed to explore the effect of different types of invitation letter on participation,^8^^,^^9^ whereas the second aimed to investigate the effect of the timing of when the SCQ was given to the participant on their completion and return rates.^10^^,^^11^ Within the invitation letter of the RCT, sample households invited to take part in NICOLA were randomly assigned to 1 of 12 variations of the invitation letter. The aim was to assess whether the signatory of the letter, the descriptor of NICOLA as a ‘study’ or a ‘project’ and the inclusion or exclusion of a statement of confidentiality within the wording of the letter affected uptake in study participation. In the SCQ RCT, participants were randomized to receive the questionnaire at the end of their home interview or posted to them separately post-interview. The aim was to examine the impact of varying the point at which the participant was issued a questionnaire on completion rates. Regardless of the way in which the SCQ was issued (i.e. at the end of the home interview or by post), the participant then completed the SCQ in their own time and posted it back to the NICOLA research office using a pre-paid envelope. Final analysis and reporting of both embedded RCTs will follow in due course.

## What has it found?

### Wave 1: fieldwork response rates

**Figure 1:**
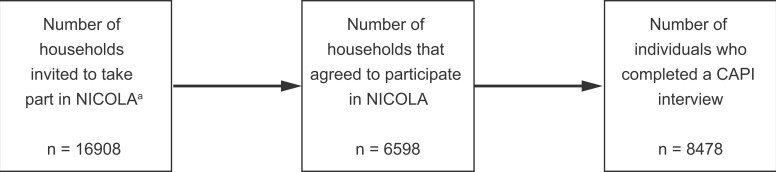
Sampling frame for NICOLA Wave 1. NICOLA, Northern Ireland Cohort for the Longitudinal Study of Ageing; CAPI, computer-assisted personal interview. ^a^Defined as people aged ≥50 years living in private residential accommodation in Northern Ireland

Of the 16 908 households who received the invitation letter to take part in Wave 1, 6598 households agreed to participate. Within these 6598 households, a total of 8478 CAPI interviews were conducted with eligible participants between December 2013 and July 2016 (see Figure 1).

Of the 8478 CAPI interviews conducted, 195 were with partners of eligible participants who were not yet 50 years of age at the time of interview. Fifty-nine percent of CAPI participants returned a valid SCQ. Table 2 shows the socio-demographic characteristics of those participants who completed the CAPI and SCQ components of the study.

**Table 2:** Characteristics of NICOLA Wave 1 total CAPI, SCQ and non-SCQ samples and comparison with Northern Ireland population aged ≥50 years^a^

		Total			Men			Women			Northern Ireland population^b^
		CAPI	SCQ	No SCQ	CAPI	SCQ	No SCQ	CAPI	SCQ	No SCQ	
		*N* = 8478	*N* = 5032	*N* = 3446	*N* = 3788	*N* = 2283	*N* = 1505	*N* = 4690	*N* = 2749	*N* = 1941	*N* = 574 215
		%	%	%	%	%	%	%	%	%	%
Sex	Men	44.7	45.4	43.7	–	–	–	–	–	–	46.8
	Women	55.3	54.6	56.3	–	–	–	–	–	–	53.2
Age group	<50	2.3	2.4	2.1	1.0	0.9	1.1	3.4	3.6	3.0	–
	50–64	49.4	50.4	47.9	48.4	47.9	49.2	50.1	52.4	46.9	54.0
	65–74	29.4	30.9	27.3	31.3	33.2	28.5	27.9	29.0	26.4	25.4
	75+	18.9	16.3	22.6	19.2	18.0	21.2	18.6	15.0	23.8	20.6
Marital status	Married/cohabiting/same-sex civil partnership	66.2	70.4	60.0	72.6	76.6	66.4	61.0	65.3	54.9	62.1
	Never married	7.8	7.1	8.9	8.7	7.3	10.9	7.1	7.0	7.4	9.7
	Separated/divorced/widowed	26.0	22.5	31.1	18.7	16.1	22.7	31.9	27.8	37.7	28.2
Living status	Living alone	25.5	22.6	29.8	21.9	18.7	26.8	28.5	25.8	32.2	Not available
	Living with others	74.5	77.4	70.2	78.1	81.3	73.2	71.5	74.2	67.8	
Education^c^	Primary/none	25.9	20.2	34.2	28.5	23.0	36.9	23.7	17.9	32.0	49.3
	Secondary	44.0	43.7	44.5	42.0	42.2	41.8	45.7	45.0	46.6	31.9
	Higher	30.1	36.1	21.3	29.4	34.8	21.3	30.6	37.1	21.4	18.8
Employment^d^	Retired	49.4	51.7	46.1	50.3	53.5	45.4	48.7	50.1	46.6	
	Employed	33.8	35.9	30.8^f^	36.0	36.4	35.3^f^	32.1	35.5	27.3^f^	
	Unemployed	2.9	2.5	3.4	3.6	3.3	4.1	2.2	1.8	2.8	Not available
	Permanently sick/disabled	7.3	5.0	10.6	7.4	4.9	11.2	7.2	5.1	10.1	
	Looking after home/family	4.7	3.8	6.2	0.7	0.5	1.0	8.0	6.5	10.1	
	In education/training/other	1.9	1.1	3.0	2.0	1.3	3.0	1.8	0.9	3.0	
Area level	1 Most deprived	19.0	16.4	22.8	18.2	16.6	20.6	19.6	16.2	24.5	
Multiple	2	19.5	18.0	21.8	18.9	17.3	21.3	20.1	18.6	22.2	Not available
Deprivation	3	21.3	20.5	22.4	21.8	20.6	23.7	20.8	20.4	21.3	
Measure^e^	4	18.4	20.7	15.1	18.3	20.1	15.6	18.5	21.1	14.8	
	5 Least deprived	21.8	24.4	17.9	22.8	25.4	18.8	21.0	23.7	17.3	
Region^e^	Urban	16.2	15.8	16.7	16.1	16.0	16.1	16.3	15.6	17.2	62.4
	Intermediate	56.5	57.7	54.8	56.4	58.7	52.9	56.7	56.9	56.3	–
	Rural	27.3	26.5	28.4	27.6	25.3	31.0	27.1	27.5	26.5	37.6

a NICOLA, Northern Ireland Cohort for the Longitudinal Study of Ageing; CAPI, computer-assisted personal interview; SCQ, self-completion questionnaire.

b Values for Northern Ireland population aged ≥50 years are based on NI Census 2011 (NISRA 2022, accessed May 2022).

Missing:

c *n* = 76 ‘don’t know’ or ‘refused’.

d *n* = 87 ‘don’t know’ or ‘refused’.

e *n* = 11 missing postcode data.

f Combines estimates for ‘Employed’ with ‘In education/training/other’ to prevent disclosure owing to low cell counts in the latter category.

There was a higher proportion of women in the CAPI sample than men (55% vs 45%). Over two-thirds of the sample were aged <70 years, with nearly half of the sample in the 50- to 64-year age group and 19% aged ≥75 years. Approximately two-thirds of participants were married or cohabiting (73% for men; 61% for women), whereas 26% were separated, divorced or widowed (19% for men; 32% for women). A quarter of the sample reported living alone. Nearly half of the sample had achieved at least secondary-level education, with 30% having a third-level qualification. A large proportion of the sample were either retired or employed (∼83% in total). The sample’s addresses were fairly evenly distributed across area-level multiple deprivation indices. More than half of the participants resided in an intermediate area (i.e. another town or city within NI), whereas just over a quarter were from rural areas and 16% were from Belfast (urban).

When comparing the characteristics of those participants who consented and took part in the CAPI but did not return an SCQ (vs those who did) (see Table 2), the following was evident (for the total sample and for men and women separately): they were more likely to be in the 50- to 64-year-old age group; to be married or cohabiting; to be living with others; to have reached secondary educational level only; to be retired; and to be living in an intermediate area (for the total sample, men and women).

### Wave 1: what has been found?

The first report from NICOLA Wave 1, utilizing CAPI data, was published in November 2017.^12^ This report focused on the following key topics: socio-demographic characteristics, labour market participation, social connectedness and engagement, lifestyle behaviours (alcohol consumption, smoking and physical activity), self-reported health, healthcare utilization and the methodology of the NICOLA study. Some of the key findings from the report are below.

The proportion of women in the sample increased with increasing age. Around two-thirds of the sample were married and <10% had never been married. Widowhood increased with increasing age—54% of women aged ≥75 years were widowed. Around one-quarter of the sample lived alone and this proportion rose to almost 50% for those aged ≥75 years. Living alone was twice as likely in the most deprived areas compared with the least deprived areas, and three times as likely in urban areas compared with rural areas. Forty-four percent of participants reported having a living parent and 22% were in the sandwich generation (i.e. having both at least one living parent and one living child). Thirty percent of those with higher education were in the sandwich generation, which is three times higher than those with no education/primary education. A quarter of participants reported that they were carers for family, friends, neighbours or others because of illness, disability or problems relating to old age. Only 2% reported that they had no one to call on if they needed help because of illness.

Men were twice as likely as women to report being in employment after the age of 65 years; women were more likely to be looking after home and/or family. As expected, better health was associated with a higher likelihood of being in work for those aged 50–74 years. There was also the anticipated gradient of increasing likelihood of being in employment with increasing levels of educational qualifications in those aged 50–64 years. This pattern was especially evident for female participants. Many of those reporting being in work after pension age were self-employed. This was particularly the case for men aged 65–74 years. Of those participants aged 50–64 years and economically inactive, 48% were retired, 36% were inactive because of illness and/or disability and the remainder looked after home and family.

Over one-third of participants reported having a limiting long-term illness. The prevalence of limiting long-term illness and limitations in activities of daily living (ADL) and instrumental activities of daily living (IADL) increased with increasing age. Women reported slightly higher rates than men of IADL limitations and slightly higher rates of fair or poor mental health. On all measures of self-reported health, married or co-habiting participants (compared with those reporting they were single, separated, divorced or widowed) reported the best health. All self-reported health measures showed a clear socio-economic gradient; in general, the excess poor health between the most and least affluent areas was ∼50%. Generally, the health of those living in the most rural areas was ∼4–5 percentage points better than those in the most urban or intermediate areas.

More than 80% of participants reported that they had visited their general practitioner at least once in the past year. Participants’ reported use of hospital outpatient clinics (46%), inpatient services (20%) and the emergency department (20%) was relatively modest. Use of secondary care (and higher-cost) services increased slightly with age. Those living in areas of the most social deprivation (compared with the least) had the highest use of each service. Those with the highest service use also reported poorer self-rated health, difficulties with ADL/IADL and/or had a limiting long-term illness.

### NICOLA Wave 2

Wave 2 of the NICOLA study was conducted between May 2017 and November 2019. All living participants who took part in Wave 1 were sent a letter inviting them to take part. Wave 2 consisted of a face-to-face CAPI. Respondents were also invited to complete a SCQ.

The questions in the CAPI and SCQ were largely based on the questions asked in Wave 1 with the addition of some new and amended questions as summarized in Table 1. Following the CAPI interview, participants were mailed an SCQ including a pre-paid envelope for returning the SCQ once completed. Unlike Wave 1, there was no health assessment at Wave 2. Although every effort was made to carry out a face-to-face interview with the participant, there were some participants in Wave 2 who were deemed unable to participate in the CAPI in person due to a physical or cognitive impairment and therefore a proxy interview was carried out with a consenting family member. Proxy interviews were only introduced into the study protocol at Wave 2 and were not conducted in Wave 1. End-of-life interviews (with relatives, friends or carers of a core NICOLA participant who had died since participating in Wave 1) were also introduced into the study at Wave 2.

A refresh of the NICOLA sample is currently being conducted as a final part of Wave 2 with the aim to recruit ∼700 new participants (representing the age category 50–54 years). A further add-on to Wave 2 has been the mailing (in February 2021) of a SCQ dealing with participants’ experiences during the COVID-19 pandemic. This specific questionnaire was designed to capture information relating to physical and mental health, food provision, financial security, work and finances, volunteering and caring, health and lifestyle, social connections, pensions and retirement during the period of COVID-19. The questionnaire content is closely harmonized with that of ELSA (https://www.elsa-project.ac.uk/covid-19-survey-content) and other UK-based cohorts (https://bristol.ac.uk/alspac/researchers/wellcome-covid-19/) contributing to the UK Longitudinal Linkage Collaboration (UK LLC)^13^ to facilitate comparative analysis. The data obtained will enable us to make pre- and post-COVID comparisons as well as UK comparisons in relation to the impact of COVID-19 on the health and wellbeing of older adults.

### Wave 2: fieldwork response rates

Of the original (*n* = 8478) Wave 1 respondents, 6152 participants took part in Wave 2 representing an overall response rate of 73%. This response rate was calculated as the percentage of Wave 2 CAPI interviews that were successfully obtained from the original Wave 1 respondents. In total, 72 proxy interviews and 548 end-of-life interviews were completed.

Table 3 shows the response rates and socio-demographic characteristics of those who participated in Wave 2. CAPI response rates at Wave 2 were similar to those of Wave 1. Consistently with Wave 1, a higher proportion of women participated in Wave 2 compared with men (55% vs 45%). Three-quarters of the sample were aged <75 years, with 41% of the sample in the 50- to 64-year age group and almost one-quarter (24%) aged ≥75 years. Approximately two-thirds of participants were married or cohabiting (72% for men; 60% for women), whereas 27% were separated, divorced or widowed (20% for men; 32% for women). Just over a quarter of the sample reported living alone. Nearly half of the sample had reached at least secondary-level education, with just over a third having a third-level qualification. The sample were fairly evenly distributed across area-level multiple deprivation indices, thus allowing comparisons with the NI population. More than half of the participants resided in an intermediate area (i.e. another town or city within NI), whereas just over a quarter were from rural areas and 16% were from Belfast (urban).

**Table 3:** NICOLA Wave 2 CAPI response rates by age and sex^a^

		Total	Men	Women
		CAPI	CAPI	CAPI
		*N* = 6152	*N* = 2765	*N* = 3387
		%	%	%
Sex	Men	44.9	–	–
	Women	55.1	–	–
Age group	<50	0.8	0.2	1.3
	50–64	41.3	38.8	43.4
	65–74	33.5	35.5	31.9
	75+	24.4	25.5	23.5
Marital status	Married/cohabiting	65.5	72.1	60.1
	Never married	7.9	8.1	7.7
	Separated/divorced/widowed	26.6	19.8	32.1
Living status	Living alone	26.0	22.4	28.9
	Living with others	74.0	77.6	71.1
Education	Primary/none	22.7	25.7	20.2
	Secondary	43.4	41.6	44.8
	Higher	33.9	32.7	34.9
Employment	Retired	60.4	61.1	59.9
	Employed	29.0	30.2	28.0
	Unemployed	1.3	1.4	1.2
	Permanently sick/disabled	5.7	5.9	5.6
	Looking after home/family	2.5	0.4	4.3
	In education/training/other	1.0	1.1	1.0
Area level	Most deprived	18.0	17.8	18.2
Multiple	2	19.0	17.8	20.0
Deprivation	3	21.2	22.0	20.5
Measure	4	18.9	18.7	19.1
	Least deprived	22.9	23.7	22.1
Region	Urban	15.5	15.2	15.8
	Intermediate	56.5	56.9	56.2
	Rural	28.0	27.9	28.0

a NICOLA, Northern Ireland Cohort for the Longitudinal Study of Ageing; CAPI, computer-assisted personal interview; SCQ, self-completion questionnaire.

Values for the self-completion questionnaire are not presented as data entry is ongoing.

Forthcoming findings from Wave 2 will provide a more in-depth analysis and document how the lives of older adults in NI have changed over the intervening period between Wave 1 and Wave 2 (i.e. 2017–2019) with a particular focus on changes in economic circumstances, physical and behavioural health, health and social care utilization and quality of life.

The study aims are broad, designed to harmonize as far as possible with those of TILDA and ELSA, and its long-term research goals are to investigate the determinants of retirement behaviour and economic wellbeing; the impact of cognitive function and sensory disability on decision-making; the determinants of disability trajectories and morbidity compression across socio-economic groups and the influence of social participation on these; and the interaction of genetic, biological and psychosocial determinants on health and mortality (see Table 1).

### Data linkage in NICOLA

The NICOLA study aims to maximize its impact through the use of administrative data linkage. All NICOLA participants are flagged on the National Health Applications and Infrastructure Services (NHAIS), which receives regular updates from the General Register Office on fact and cause of death; participants’ registered address information is used to identify when a NICOLA cohort member has died. NICOLA utilizes a record linkage flag within HSC administrative data via the BSO HBS^5^ for mortality data of participants. As of the end of March 2021, there were 924 deaths among NICOLA participants. Flagging with NHAIS will enable wider anonymized linkages for research, employing safe-haven research conditions. Permissions are currently being sought for linkage of NICOLA data to routinely collected health and social care data from the BSO and HBS^5^, and also linkage with the Northern Ireland Cancer Registry^6^. Examples of such data include primary and secondary care data including hospital episode statistics, emergency department admissions and the Enhanced Prescribing Database, which will provide information on medications dispensed in community pharmacies for NICOLA participants.

In March 2021, NICOLA also became one of many studies contributing to a national research partnership the UK Longitudinal Linkage Collaboration (UK LLC)^13^, which is embedded within the COVID-19 Longitudinal Health and Wellbeing National Core Study (part of the national COVID-19 study portfolio). The UK LLC has been designed to allow large population-based studies such as NICOLA to fully contribute to the national research programme and policy development in response to the COVID-19 pandemic. It brings together data from UK-based longitudinal studies and manages the linkage and integration of data to routine health and administrative records, thus providing greater breadth and depth of data, and enabling researchers to answer priority research questions to help inform government policy and healthcare decisions.

## What are the main strengths and weaknesses?

Strengths of NICOLA are in its large sample size and the in-depth face-to-face questionnaire and supporting SCQ, the scope and detail of which allow us investigate trajectories of ageing in Northern Ireland and their biological and social determinants, including the Troubles. The value of NICOLA is also being magnified through its data linkages. The impact of NICOLA is growing through its scientific outputs and dissemination activities, which are developing year on year. Alongside this, co-production and dissemination of reports and newsletters with and to the participants are integral to the study, alongside the development of the study website (https://nicola.qub.ac.uk/).

A limitation, common to many of the ageing cohort studies, is in the self-reported nature of data; furthermore we are limited to participants living in private residences. There is also an acknowledged response bias in terms of over-representation of those with higher education levels. The study was designed to be representative of the older adult population; however, as with all health-related surveys, we cannot rule out the possibility of selection bias in that the study may have attracted relatively healthier individuals. Although ethnic minorities are also included within the cohort, the small number included within this group in NI would not be enough to report statistically robust estimates.

## Where can I get hold of the data? Where can I find out more?

NICOLA Wave 1 and Wave 2 data are maintained and stored at a repository within the Centre for Public Health, Queen’s University Belfast. Approved researchers and others from the practitioner and policy communities who wish to access the anonymized data set from the first wave survey can do so by making an application using the designated Research Proposal Form available on the study website.^14^ Supporting meta-data documentation including, but not limited to, data dictionaries, derived variables and data access policies can also be accessed via the NICOLA website at https://www.qub.ac.uk/sites/NICOLA/InformationforResearchers/. At present, data access is via the NICOLA safe setting within the Centre for Public Health, Queen’s University Belfast. Core variables are also archived on an ongoing basis with the UK Data Service^15^ and the UK LLC^13^. In the longer term, the NICOLA team also plans to archive core variables via the Dementias Platform UK^16^. It is also the intention of the study team to collaborate with the data-sharing and harmonization initiative, the Gateway to Global Aging Data (G2G)^17^, thus facilitating cross-country research by identifying comparability between NICOLA and other ageing cohort studies.

## Ethics approval

The NICOLA study is sponsored and ethically reviewed and approved via the School of Medicine, Dentistry and Biomedical Sciences of Queen’s University Belfast (Ref: 12/23). Participants provided written informed consent prior to participation in the study.

## Data Availability

See ‘Where can I get hold of the data?’ above.
